# High-Quality Genome Assembly and Transcriptome of *Rhododendron platypodum* Provide Insights into Its Evolution and Heat Stress Response

**DOI:** 10.3390/plants14081233

**Published:** 2025-04-17

**Authors:** Zizhuo Wang, Kunrong Qin, Wentao Chen, Guanpeng Ma, Yu Zhan, Haoxiang Zhu, Haiyang Wang

**Affiliations:** 1 College of Horticulture and Landscape Architecture, Southwest University, Chongqing 400715, China; wzz562599702@email.swu.edu.cn (Z.W.); qoraqin123@email.swu.edu.cn (K.Q.); 19196302270m@sina.com (W.C.); guanpengma@163.com (G.M.); zhanyu02@126.com (Y.Z.); zhuhx8910@swu.edu.cn (H.Z.); 2 School of Architecture and Design, Chongqing College of Humanities, Science & Technology, Chongqing 401524, China; 3 Horticulture Institute, Guizhou Academy of Agricultural Sciences, Guizhou 550006, China

**Keywords:** evolution, heat stress, high-quality genome assembly, rocky habitat adaptation, transcriptome

## Abstract

*R. platypodum* (*Rhododendron platypodum*) is an endangered alpine species with a highly restricted distribution in the southwestern region of China, which possesses significant ornamental and horticultural value. In this study, the high-quality genome assembly of *R. platypodum* at the chromosomal level is reported. The total genome size was determined to be 642.25 Mb, with a contig N50 of 25.64 Mb, and it contains 36,522 predicted genes. Comparative genomic analysis between *R. platypodum* and other species revealed the expansion of gene families, such as those related to transition metal ion binding and sodium ion transport, as well as the contraction of gene families involved in the recognition of pollen and pollen–pistil interaction. These findings might explain the adaptation of *R. platypodum* to rocky habitats and contribute to its endangered status. Furthermore, a heat stress experiment was conducted on *R. platypodum*, followed by transcriptome sequencing and physiological co-analysis to construct a co-expression network. This analysis identified the candidate gene *TAR1-A* and other transcription factors exhibiting differential expression under heat stress. The whole-genome sequencing, transcriptome analysis, and physiological co-analysis of *R. platypodum* provide valuable resources for its conservation and offer insights into its mechanisms of heat stress.

## 1. Introduction

Rhododendrons are important horticultural and ornamental plants worldwide, valued for their large, vibrant flowers and graceful forms [1,2,3]. The genus *Rhododendron* encompasses 600 confirmed species, making it the genus with the highest number of woody plant species in China [4]. Taxonomists have classified the genus into eight subgenera based on morphological characteristics, with *Hymenanthes* recognized as being widely distributed in high-altitude areas [5]. However, urban development and global climate change have significantly impacted high-altitude habitats, placing *Hymenanthes* species at risk of extinction [4,6]. *R. platypodum*, a typical representative of this group, is an evergreen shrub, primarily found in rocky and forested areas at altitudes of 1800–2100 m [7]. Due to factors such as human activities and its low adaptability, the habitat of *R. platypodum* has been severely reduced, with its distribution now confined to a small area on steep slopes and rocks in southeast Chongqing and the Mao’er Mountains in Guangxi, China. As a result, it has been included in the Red List of Rhododendrons [8] and highlighted by the International Union for Conservation of Nature (IUCN) [9]. Despite its high ornamental value, attributed to its large, variably colored flowers (white, pink, and purple) and elegant form, research on *R. platypodum* remains limited. Current studies primarily focus on its genetic diversity, phenotypic diversity, and chloroplast genome [7,9]. Further research is necessary to promote its conservation and enhance its ornamental utilization.

With the advancement of sequencing technologies, over 10 species within the genus *Rhododendron* have undergone complete genome assembly, including species from subgenera such as *Hymenanthes*, *Pentanthera*, *Tsutsusi*, and *Azaleastrum* [10,11,12,13]. Species with high environmental adaptability, such as *Rhododendron simsii* (*R. simsii*) and *Rhododendron ovatum* (*R. ovatum*) [10,11], and those with more restricted distributions, such as *Rhododendron henanense* (*R. henanense*) and *Rhododendron griersonianum* (*R. griersonianum*), have also been sequenced [1,4]. Despite these advancements, genomic research remains limited, considering the 600 confirmed species within the genus. The genomic study of *R. platypodum* could provide a more comprehensive genomic resource, benefiting both the conservation and development of *Rhododendrons*.

Species within the subgenus *Hymenanthes* are predominantly distributed in high-altitude regions and typically exhibit poor heat tolerance [14]. In the context of global warming, current research on the heat tolerance of *Rhododendrons* mainly focuses on other subgenera or widespread species capable of withstanding high temperatures [10,15]. Studies have examined physiological [16,17], biochemical [15], and transcriptomic [14,18,19] changes and response mechanisms to heat stress. Yet there is limited research linking physiological and transcriptomic data through co-expression network analysis. Weighted correlation network analysis (WGCNA), a statistical and bioinformatic method, can help elucidate the molecular mechanisms underlying heat stress in plants and provide candidate genes for the breeding of heat-tolerant varieties [20,21].

In this study, we report the high-quality genome assembly of *R. platypodum* and construct a phylogenetic tree by comparing it with other species. This comparison reveals genomic changes throughout its evolutionary process. Furthermore, we integrate physiological and transcriptomic analyses to assess the adaptive mechanisms of *R. platypodum* under high-temperature conditions. This study will lay the foundation for the future conservation, low-altitude introduction, and domestication of *R. platypodum*.

## 2. Results

### 2.1. Genome Assembly and Assessment

The genome assembly of *R. platypodum* was generated from fresh leaf tissues of a diploid individual collected from Jinfoshan Mountains, Chongqing (107°11.38′ E, 29°02.42′ N). High-molecular-weight DNA was extracted using an optimized CTAB method and sequenced with PacBio Revio HiFi (long-read, 31.8× coverage) and BGI Illumina (short-read, 150.3× coverage) platforms. De novo assembly was performed using HiFiasm v0.16.1, followed by Hi-C scaffolding to cluster contigs into chromosome-scale scaffolds.

K-mer analysis estimated the genome size to be 630.6 Mb (Figure S1). The final assembly spanned 642.3 Mb (contig N50: 25.6 Mb) with 98.8% core eudicot BUSCO completeness (93.2% single-copy, 5.6% duplicated; Table S1), surpassing existing Rhododendron genomes (Table 1). HiFi and Illumina read mapping rates reached 99.8% and 98.9%, respectively, with coverage rates >99.8% (BWA/Minimap2). The assembly quality (QV: 65.2) and LAI score (21.1, “golden” level) further validated its integrity. The Hi-C sequencing (Figure S2; Table S2) supported a near-chromosome-level assembly, with 13 chromosome-scale scaffolds (636.9 Mb, 99.2% of total length; scaffold N50: 493.6 Mb) matching the expected diploid karyotype (2n = 26).

### 2.2. Genome Annotation of R. platypodum

A comprehensive comparison across multiple databases resulted in the annotation of a total of 36,522 genes in the *R. platypodum* genome, of which 35,679 genes (97.7%) were successfully annotated (Table S3). Following genome annotation, the average gene length was determined to be 6460 bp, with an average coding sequence length of 1132 bp, an average exon length of 367 bp, and an average intron length of 1194 bp (Table 1 and Table S4, Figure S3). Non-coding RNAs were also annotated, revealing 87 miRNAs, 649 tRNAs, 1964 rRNAs, and 480 snRNAs, which together accounted for 1.33% of the total genome (Table S5).

Notably, a comparison with the genomes of 10 other *R.* species (Table 1) revealed that *R. platypodum* has the highest average gene length (6460 bp), indicating a higher level of genomic complexity. And *R. platypodum* exhibited the highest percentage of repeat sequences among the genus *Rhododendron* species (69.6%). *R. platypodum* demonstrated a high proportion of LTR retrotransposons (47.7%), second only to *R. henanense* (50.8%). This might be related to its relatively long average genome length and the presence of gene duplication sequences.

### 2.3. Phylogenetic and Gene Family Expansion and Contraction Analysis

**Figure 1:**
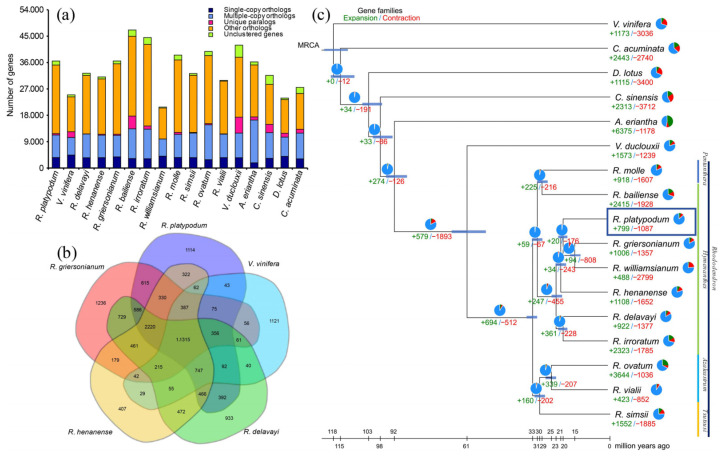
(**a**) Bar graph showing the species-specific or nonspecific properties of all genes in 17 species. (**b**) The Venn diagram shows the distribution of shared or unique orthogroups among five Rhododendron species. (**c**) Phylogenetic tree showing the topology and divergence times for 19 plant species. Divergence times are indicated by light blue bars at the internodes. The range of the light blue bars indicates the 95% highest posterior density of the divergence time. Numbers in green and red at branches indicate the expansion and contraction of gene families, respectively. The pie chart colors represent the changes in gene families (blue—no significant change; orange—expanded or contracted; green—expanded; red—contracted); each color sector illustrates the proportion of each type of gene family change. Note: refer to Table S6 for the standardized abbreviations of plant species names.

The genome of *R. platypodum* was compared with those of 16 other plant species, all of which were classified under the ARP III classification system. These species were selected based on different subgenera of the *Rhododendron* genus, different genera within the Ericaceae family, and different families within the Ericales order. An outgroup, *Vitis vinifera* (*V. vinifera)*, was also included (Table S6). A total of 19,122 gene families were identified in *R. platypodum*, of which 101 were unique to this species (Figure 1a; Table S7). *R. platypodum* shared more specific gene families with *R. griersonianum* (615) than with more distantly related species such as *R. henanense* (322) and *Rhododendron delavayi* (*R. delavayi*) (194) (Figure 1a,b).

According to the phylogenetic tree, the evolutionary history of *R. platypodum* could be traced (Figure 1c). The divergence of the families Diospyros (*Diospyros kaki*), Theaceae (*Camellia sinensis*), and Actinidiaceae (*Actinidia eriantha*) within the order Ericales occurred at 102.4 Ma, 97.6 Ma, and 91.8 Ma, respectively. In the Ericaceae family, the divergence between the genera *Vaccinium* (*Vaccinium duclouxii*) and *Rhododendron* occurred at 60.7 Ma, accompanied by the significant expansion and contraction of gene families (+597 and −1893, respectively). Within the genus *Rhododendron*, gene families underwent intense expansion and contraction (+694 and −512, respectively) at 32.4 Ma, leading to differentiation into evergreen and deciduous rhododendrons (subgenus *Azaleastrum* and subgenus *Tsutsusi*). *Rhododendron platypodum* (*R. platypodum*) belongs to the subgenus *Hymenanthes*, diverging at 21.8 Ma from *R. delavayi* and *Rhododendron irroratum* (*R. irroratum*), and at 18.8 Ma from *R. griersonianum* and *Rhododendron williamsianum* (*R. williamsianum*). Notably, *R. platypodum* exhibited fewer gene family expansions and contractions compared to other subgenus Hymenanthes species (+799 and −1087, respectively).

**Figure 2:**
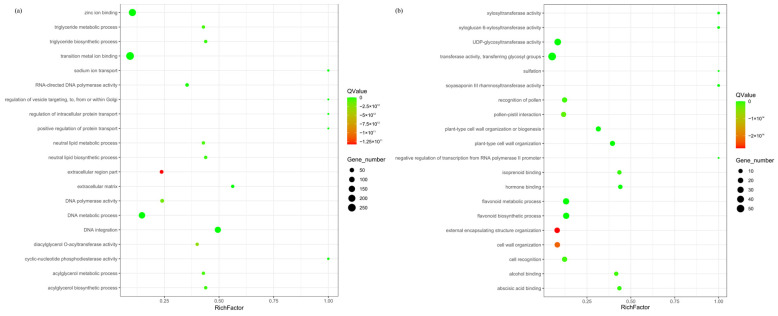
Enrichment analysis of expanded (**a**) and contracted (**b**) gene families in *R. platypodum*.

The expanded and contracted gene families of *R. platypodum* were annotated by Gene Ontology (GO). Among the expanded gene families (Figure 2a), the most enriched categories in the molecular function category were binding (839) and catalytic activity (804) (Figure S4a). The most abundant terms included transition metal ion binding, zinc ion binding, DNA metabolic process, and DNA integration (*p* < 0.05). Within the contracted gene families (Figure 2b), the molecular function category showed the highest enrichment for catalytic activity (171), and the Cellular Component category showed the highest enrichment for membrane (141) (Figure S4b). The most significant terms included flavonoid metabolic process, flavonoid biosynthetic process, transferase activity, transferring glycosyl groups, and UDP-glycosyltransferase activity.

### 2.4. Differential Expression of Physicochemical Properties and Genes in R. platypodum Under Heat Stress

Leaf samples from three-year-old Rhododendron platypodum plants exposed to 36 °C/28 °C (day/night) heat stress for 0 (CK), 1, 2, 4, and 6 days were subjected to RNA-Seq and an analysis of physicochemical properties. Due to the thick leaf cuticular wax of *R. platypodum*, no significant wilting was observed during heat stress, and only noticeable curling was observed (Figure S5). After the onset of heat stress, the stomatal W/L ratio and size of *R. platypodum* significantly increased at 1d (Figure S6a,b). After stabilizing for a period, both parameters increased significantly again at 6d. Fv/Fm and relative conductivity exhibited a continuous decline during heat stress, but the changes were not significant (Figure S6c,d).

**Figure 3:**
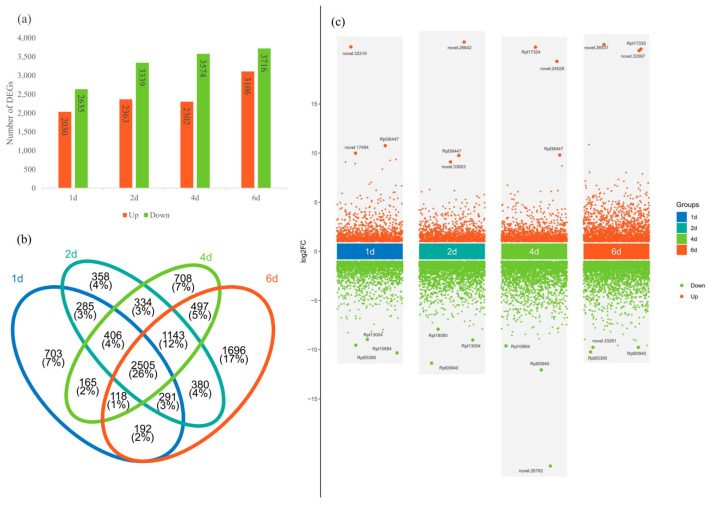
(**a**) The number of upregulated or downregulated DEGs in different treatment groups in *R. platypodum*. (**b**) The number of shared or unique DEGs across different treatment groups. (**c**) Volcano plots of DEGs in treatment groups.

Total RNA was extracted from three biological replicates per treatment and sequenced on the BGI PE150 platform. After the filtering of low-quality reads and adapters, clean reads were mapped to the *R. platypodum* genome, and the number of FPKM was calculated for each gene. Principal Component Analysis (PCA) was performed to analyze the control group (0d) and treatment groups (1d, 2d, 4d, and 6d) (Figure S7). The results indicated that the samples within each group clustered effectively, and there was a significant difference between the control and treatment groups. The 1d, 2d, and 4d groups showed relatively tight clustering, while the 6d group was more distinct, similar to the physiological response of *R. platypodum* (Figure S6). In a comparison of multiple treatment groups with the control group, 4655 differentially expressed genes (DEGs) were found in the 1d vs. 0d comparison (2030 upregulated and 2635 downregulated), 5702 DEGs in the 2d vs. 0d comparison (2365 upregulated and 3339 downregulated), 5876 DEGs in the 4d vs. 0d comparison (2302 upregulated and 3574 downregulated), and 6822 DEGs in the 6d vs. 0d comparison (3106 upregulated and 3716 downregulated) (Figure 3a). A total of 2505 DEGs (26%) were common across all comparisons, with the 6d vs. 0d comparison having the largest number of unique DEGs (1696) (Figure 3b). We found that the three most upregulated DEGs at each time point included eight genes annotated as *TAR1-A* genes (*novel.33319*, *Rpl36447*, *novel.17494*, *novel.26642*, *Rpl36447*, *novel.33603*, *Rpl17324*, *Rpl36447, Rpl17335*, *novel.26537*, *novel.33597*) (Figure 3c). A functional enrichment analysis of the DEGs in the four treatment groups revealed several common enriched pathways, including environmental response signaling pathways (ligand-gated ion channel activity and signaling receptor activity) and enzyme-related metabolic pathways (protein kinase activity and protein serine kinase activity) (Figure S8).

**Figure 4:**
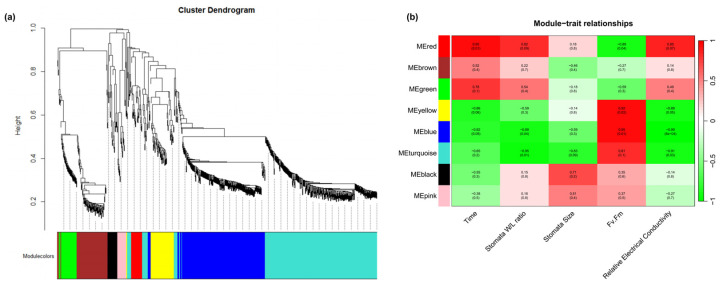
Transcriptomic correlation analysis across physiological index and heat stress time in *R. platypodum*. (**a**) Dendrogram showing co-expression modules (clusters) identified by weighted correlation network analysis (WGCNA) across physiological index and heat stress time. The major tree branches constitute 8 modules labeled with different colors. (**b**) The heat map shows the correlation between physiological index and modules. Red color indicates a positive correlation between the cluster and the tissue. Green color indicates a negative correlation.

To further investigate the regulatory mechanisms underlying the physiological changes in *R. platypodum* during heat stress, weighted gene co-expression network analysis (WGCNA) was performed. A total of eight co-expression modules were identified (Figure 4a). The turquoise module exhibited correlation with the stomata W/L ratio, stomata size, and relative conductivity, while the blue module showed correlation with the stress time, stomata W/L ratio, Fv/Fm, and relative conductivity. These two modules might be involved in the physiological changes in *R. platypodum* under heat stress (Figure 4b). An enrichment analysis of the genes in the blue and turquoise modules revealed that the most significant terms were response to stress, response to stimulus, and response to chemical, further supporting the association of these modules with heat stress (Figure S9).

**Figure 5:**
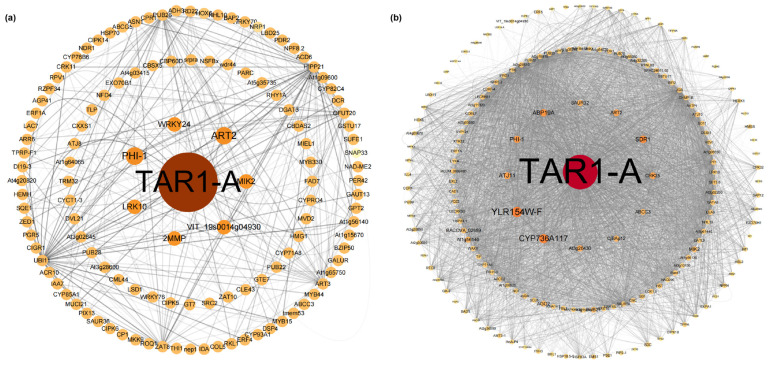
(**a**) Blue module co-expression network; (**b**) turquoise module co-expression network.

The blue and turquoise modules were selected to construct a visual network in Cytoscape (Figure 5). Similarly to the results from DEG analysis (Figure 3b), *TAR1-A* also played a dominant role in the co-expression network, with its expression showing a trend of downregulation followed by upregulation (Table S8). In the hub genes of both modules, we identified genes related to ABA biosynthesis and signaling pathways (*PHI-1*, *SDR1*, *CRK25*, *LRK10*), transcription factors associated with ABA biosynthesis and signaling (*WRKY24*), and genes involved in biotic and abiotic stress (*MIK2*, *ABCC3*, *CJBAP12*). These genes generally exhibited a downregulation trend (Table S8). Genes related to auxin biosynthesis, such as *ABP19A* and *SAUR32*, showed upregulation and downregulation trends, respectively.

### 2.5. Transcription Factor Expression Analysis

**Figure 6:**
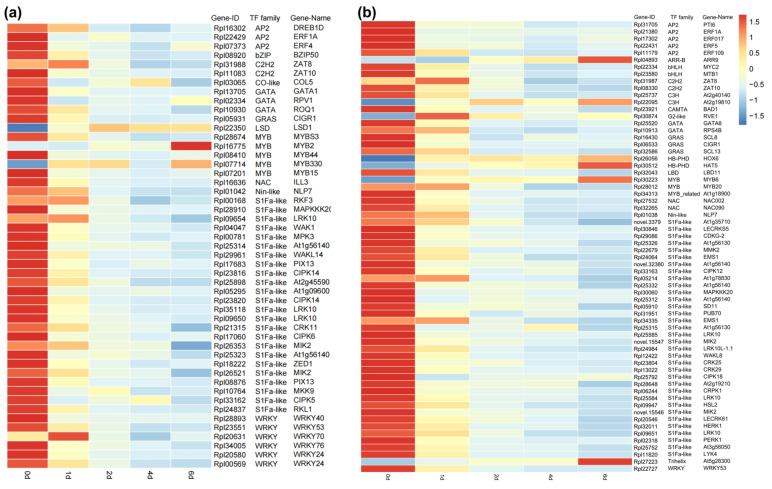
The expression levels of transcription factors and their corresponding gene families in the two modules. (**a**) Blue module; (**b**) turquoise module.

In the blue module, a total of 49 transcription factors (TFs) were identified, while 64 TFs were identified in the turquoise module. These TFs belonged to families such as *S1Fa-like*, *WRKY*, *MYB*, and *GATA* (Figure 6). Gene expression patterns of differentially expressed TFs were visualized using a heatmap, which revealed that most genes exhibited the highest expression at day 0 and showed a continuous downregulation trend. Genes such as *ZAT8*, *RKF3*, *LRK10*, *WRKY70*, *RVE1*, *LBD11*, *MYB20*, *At1g78830*, and *EMS1* showed significant upregulation at day 1, but subsequently displayed a decline. Only a few genes, including *ARR9*, *At2g19810*, *HOX6*, *HAT5*, *MYB6*, *MYB2*, *At5g28300*, and *LSD1*, demonstrated a sustained upregulation trend.

## 3. Discussion

In this study, we presented a high-quality, chromosome-level whole-genome assembly of *R. platypodum*. The LAI value and the complete BUSCOs were among the highest reported in recent Rhododendron genome studies (Table 1), providing a genetic foundation for the conservation and future utilization of this rare species. Additionally, a heat stress experiment was conducted on *R. platypodum*, and candidate genes involved in its heat stress response were identified through weighted gene co-expression network analysis (WGCNA). These findings offer valuable insights into the potential mechanisms of heat tolerance in *R. platypodum*.

### 3.1. Unique Genetic Features of R. platypodum

The genomic repeat sequences in *R. platypodum* were larger than those found in any previously studied Rhododendron genome, comprising 69.63% of its total genome. The average gene length was also longer, measuring 6459.24 bp, primarily due to the high presence of transposable element (TE) sequences, with LTR retrotransposons (LTR-RTs) constituting a significant portion (Table 1). The elevated TE content might be linked to extensive hybridization events in the subgenus *Hymenanthes* in the southwestern region [26], which could have activated the activity of LTR-RTs [22,27]. These genomic features were similar to those observed in *R. henanense*, and might be related to their unique adaptability. It is precisely this uniqueness that has led to their limited distribution range and endangered status [1].

In our study, we focused on assembling and analyzing the nuclear genome of *R. platypodum.* The chloroplast genome of this species has been previously published [9]. However, the mitochondrial genome has not yet been characterized. Plant mitochondrial genomes are known for their considerable variability in size and structure, posing significant challenges for assembly and analysis. Additionally, distinguishing mitochondrial sequences from potential bacterial contaminants can be difficult [28]. Future studies employing advanced sequencing technologies and specialized assembly tools will be necessary to elucidate the mitochondrial genome of *R. platypodum*, thereby enhancing our understanding of its genetic makeup and evolutionary adaptations.

### 3.2. Unique Evolutionary Characteristics of R. platypodum

In the subgenus Hymenanthes, *R. platypodum* exhibited the least gene family expansion and contraction (Figure 1c), which might be attributed to its stable and specific habitat, resulting in a highly conserved and stable genome [29]. Previous studies have also shown that the expansion of specific functions in *Rhododendrons* is induced by environmental factors [10,22]. The expansion and contraction of gene families in *R. platypodum* are relatively unique. Compared to other species, *R. platypodum* showed gene family expansions in categories such as zinc ion binding, transition metal ion binding, and sodium ion transport. This might be linked to its distribution in the karst regions of southwestern China, where soils and rocks typically contain high levels of heavy metals, particularly zinc [30,31]. These gene family expansions could facilitate the adaptation of *R. platypodum* to heavy metal-rich rocky habitats (Figure 2a) [7]. And significant reductions in gene families related to pollen recognition and pollen–pistil interactions were observed in *R. platypodum* (Figure 3b). This contraction might be induced by the heavy metal-rich habitat [32] and could contribute to its endangered status [33]. Furthermore, several gene families involved in glycosyltransferase activities were annotated (e.g., xylosyltransferase activity, XYLOGlucan 6-xylosyltransferase activity, UDP-glycosyltransferase activity, transferase activity, transferring glycosyl groups), which could reduce the diversity of small molecules in *R. platypodum*. These effects might negatively impact processes such as seed germination, growth, flowering, and fruiting [34,35].

### 3.3. Coordinated Analysis of Physiology and Transcriptome in R. platypodum Under Heat Stress

The leaves of plants could indirectly reflect their ability to cope with heat stress [36]. In our study, as the duration of heat stress increased, the relative conductivity, Fv/Fm, stomata W/L ratio, and stomata size consistently exhibited upregulation or downregulation (Figure S6), indicating a significant response of *R. platypodum* to heat stress. Similar responses have been reported in other *Rhododendrons* [16]. *R. platypodum* did not exhibit significant yellowing or scorching during prolonged heat stress but showed noticeable leaf curling (Figure S5). This might be linked to sustained stomatal expansion (Figure S6a,b), which facilitated cooling through continuous transpiration [37]. The reduction in water content and the resulting accumulation led to leaf curling [38,39].

Subsequently, we employed weighted gene co-expression network analysis (WGCNA) to identify candidate genes associated with heat stress. After annotating the genes in the two identified modules, we found correlations with terms such as response to stress, response to stimulus, and response to chemical, confirming the association of these modules with heat stress (Figure S9). Both differentially expressed genes (DEGs) and WGCNA revealed that the *TAR1-A* gene played a dominant role (Figure 3c and Figure 4). In *Arabidopsis thaliana* and *Artemisia caruifolia*, the *TAR1-A* gene has been shown to increase cuticular wax thickness and regulate trichome development [40]. In our study, the expression of *TAR1-A* exhibited a characteristic pattern of first decreasing and then increasing under heat stress (Table S7). It is hypothesized that it might initially reduce the accumulation of leaf cuticle thickness and enhance stomatal conductivity to facilitate heat dissipation [41], reflected in the significant increase in stomatal aperture and area on day 1 (Figure S6a,b). Furthermore, we identified genes related to abscisic acid (ABA) synthesis and signaling pathways in the co-expression network (*PHI-1*, *SDR1*, *CRK25*, *LRK10*), which showed continuous downregulation during heat stress (Table S7). These genes likely cooperate to maintain stomatal opening [42,43]. As heat stress progressed and the leaves continued to lose water and curl, the expression of *TAR1-A* was upregulated on day 6, possibly reducing non-stomatal water loss by decreasing the cuticular wax [44,45]. In conclusion, *TAR1-A* might play a crucial role in the heat tolerance of *R. platypodum*, but further studies are needed to elucidate its genetic function and underlying mechanism.

### 3.4. Transcription Factors in R. platypodum Under Heat Stress

To further investigate the role of transcription factors in *R. platypodum* heat stress responses, we identified the transcription factors of genes from two modules. The major transcription factor families identified include *S1Fa-like*, *WRKY*, *MYB*, and *GATA* (Figure 6). The *S1Fa-like* family was the most abundant and exhibited a consistent decline, which aligns with the response of cotton to various abiotic stresses [46]. However, research on this family is still limited and requires further exploration. Among the hub genes in the blue co-expression network, we identified a consistently downregulated transcription factor, *WRKY24* (Figure 5a and Figure 6a). Previous studies have shown that the transient silencing of *WRKY24* can increase wheat’s sensitivity to drought stress [47], and *WRKY24* expression is also downregulated in rice under drought and salt stress [48]. This transcription factor might contribute to the heat sensitivity of *R. platypodum*. Among the upregulated transcription factors, we identified two *MYB* family genes (*MYB2* and *MYB6*). Previous reports indicate that the upregulation of *MYB6* and *MYB2* has been shown to enhance the expression of flavonoid biosynthesis genes in *Populus* and *Dendrobium bigibbum*, leading to the significant accumulation of anthocyanins and proanthocyanidins [49,50]. This phenylpropanoid biosynthesis is considered crucial in stress responses [51], and these *MYB* genes might serve as candidate genes for future studies on heat tolerance in rhododendrons. These findings provide valuable candidate genes for further research on heat tolerance in *R. platypodum*.

## 4. Material and Methods

### 4.1. Plant Samples, Sequencing Process, and Assembly

Fresh, clean *R. platypodum* leaves were collected from Jinfoshan Mountains, Chongqing (107°11.384413′ E, 29°02.429537′ N). After washing, the leaves were immediately frozen in liquid nitrogen and stored at −80 °C until DNA extraction. High-quality genomic DNA was extracted from the tissue using a modified CTAB method [52].

Approximately 15 μg of genomic DNA was sheared into 15 kb fragments using the Covaris g-TUBE (Covaris LLC, Woburn, MA, USA), and fragment size was assessed using the Femto Pulse system (Agilent Technologies, Santa Clara, CA, USA). Size selection was performed on the SMRT Bell template with the BluePippin system (Sage Science, Beverly, MA, USA), enriching for fragments larger than 15 kb. The quality and quantity of the library were evaluated using the Femto Pulse system (Agilent, Santa Clara, CA, USA) and the Qubit fluorometer (Thermo Fisher Scientific, Waltham, MA, USA). Sequencing was performed on the PacBio Revio platform (Pacific Biosciences of California, Menlo Park, CA, USA), generating 20.44 Gb (31.8×) of highly accurate (>99%) HiFi read data for genome assembly. The draft genome assembly was performed using HiFiasm (v0.16.1, default parameters) [53], and gfatools (https://github.com/lh3/gfatools, accessed on 22 September 2025) was used to convert sequence graphs in GFA to FASTA format. An Illumina library (insert fragment size: 350 bp) was also constructed and sequenced on the BGI platform (BGI Group, Shenzhen, China), generating 96.55 Gb (150.3×) of raw sequence data.

The Hi-C sequencing produced 76.48 Gb of read data (119.1×). Then, high-quality paired-end Hi-C reads were processed with Trimmomatic (v0.40) [54] to remove low-quality bases and adapter contaminants (using parameters LEADING:3, TRAILING:3, SLIDINGWINDOW:4:15, MINLEN:15). The resulting clean reads were then aligned to the pre-assembled contigs using the Juicer pipeline (v3; https://github.com/aidenlab/juicer, accessed on 27 September 2025) [55] to generate a contact frequency map. Next, two rounds of misjoin correction were carried out with 3D-DNA (v180922) [56] using default settings (–r2) to construct an initial scaffold layout. The oriented scaffolds were further processed by generating interaction matrices with Juicer and manually curated using Juicebox Assembly Tools (v1.11.08) [55]. Through the manual curation of the assembly results, we achieved a high-quality, near-chromosome-level assembly comprising 13 chromosome-scale scaffolds (consistent with the expected karyotype of *R. platypodum*, 2n = 26), totaling 636.89 Mb and accounting for 99.17% of the total assembly length, with a scaffold N50 of 493.61 Mb (Table S8).

### 4.2. Assembly Quality Assessment

A comprehensive assessment of the *R. platypodum* genome was conducted. First, the completeness of the genome assembly was evaluated using Benchmarking Universal Single-Copy Orthologs (BUSCO, v3.0.2) [57], which assesses the presence of conserved core plant genes in the *R. platypodum* genome. The remapping rate and coverage were evaluated using both Illumina and long reads. Illumina short reads were aligned using BWA MEM (v0.7.17) software, while long reads were aligned with Minimap2 (v2.24) [58], utilizing the “-ax map-ont/map-hifi” parameter. To assess complex repetitive sequences, the Long Terminal Repeat (LTR) Assembly Index (LAI) [59] was calculated, and the LAI value for LTR elements was estimated. Additionally, k-mer-based quality assessment was performed using the Merqury pipeline (v1.3) [60] and HiFi reads (k = 19 bp).

### 4.3. Repeat Annotation

Repetitive sequences, including tandem repeats and transposable elements (TEs), were annotated using Tandem Repeats Finder (TRF, v4.09.1) with the following parameters: 2 7 7 80 10 50 2000 [61]. TEs were annotated using a combination of de novo and homology-based methods. LTR_FINDER (v1.0.7) was used to identify LTR retrotransposons (LTR-RTs) [62], and RepeatModeler (v2.0.1) was used to build a de novo repeat library [63]. RepeatMasker (v4.1.2) was then used to search known TEs in the Repbase library and the de novo library [64,65,66]. RepeatProteinMask (within the RepeatMasker package) was used to search TE proteins at the protein level using the WU-BLASTX engine (v2.0).

### 4.4. Gene Annotation

Gene structure was predicted using homologous, ab initio, and transcriptome-assisted annotation. Tblastn (v2.11.0+) was used for homologous alignment to related species, and Exonerate (v2.4.0) was used for precise alignment [67,68]. Augustus (v3.4.0) and GlimmerHMM (v3.0.4) were used for de novo gene annotation [69,70,71]. All transcriptome data after heat stress within this study were used as RNA-seq data, and both genome-guided and de novo transcriptome assemblies were performed using HiSat2 (v2.2.1) for alignment and Stringtie (v2.1.7) and Trinity (v2.8.5) for transcript assembly [72,73,74]. The integrated transcriptome was built using the PASA (v2.4.1) pipeline with RNA-seq data. Finally, the gene set was refined and integrated using Maker (v3.01.03) and PASA for UTR and alternative splicing isoform annotation [75,76].

### 4.5. Functional Annotation, Non-Coding RNA Annotation, and Gene Family Identification

Gene functions were inferred by aligning sequences to databases such as National Center for Biotechnology Information Non-Redundant (NCBI NR), Kyoto Encyclopedia of Genes and Genomes (KEGG), Gene Ontology (GO), TrEMBL, and Swiss-Prot using Diamond BLASTP (v2.0.7) (E-value threshold 1 × 10^−5^) [77]. Protein domains were annotated using InterProScan (v5.50-84.0) based on the InterPro database [78,79].

Non-coding RNAs were annotated using several tools: tRNAscan-SE (v2.0.9) for tRNA [80], RNAmmer (v1.2) for Rrna [81], and Infernal (v1.1.2) against the Rfam (v14.6) database for miRNA and snRNA identification [82,83].

To identify protein-coding gene families, we used OrthoFinder (v2.5.4) [84] to cluster proteins from *R. platypodum* and 16 other species (Table S1) using the “-M msa-S diamond” parameters.

### 4.6. Phylogenetic Analysis

Single-copy orthologs were selected to construct a phylogenetic tree. Protein sequences were aligned with MUSCLE (v3.8.31) [85], and the corresponding coding sequences were aligned and concatenated based on protein alignments. The phylogenetic tree was built using the maximum likelihood method in RAxML (v8.2.12) [86]. Divergence times were obtained from TimeTree (http://www.timetree.org/, accessed on 29 September 2025) [87], and time corrections were applied using R8S (v1.81) [88]. Divergence times were further estimated with MCMCTree in PAML (v4.10.7) [89].

### 4.7. Gene Family Expansion and Contraction Analysis

Gene family expansion and contraction were analyzed using CAFÉ [90], based on the identified gene families and the phylogenetic tree with estimated divergence times. A random birth–death model was applied to calculate conditional *p*-values for each gene family. Families with a *p*-value < 0.05 were considered to have significant gene gain or loss rates. The expanded and contracted gene families were subjected to GO and KEGG enrichment analysis, using a hypergeometric test with FDR-adjusted *p*-values (Q-value) < 0.05.

### 4.8. RNA Sequencing, Gene Expression Analysis, and Physiological Measurements

Three-year-old *R. platypodum* plants, propagated from a uniform batch, were selected as experimental materials. After transplantation into a controlled environment chamber, the plants were subjected to heat stress at 36 °C/28 °C (day/night) for 0 (CK), 1, 2, 4, and 6 days. Leaf samples were collected for RNA sequencing and physiological measurements. Three biological replicates were used for each treatment. RNA was extracted and subjected to BGI PE 150 sequencing. Following sequencing, SOAPnuke (v2.1.0) was used to remove low-quality reads, N bases, and adapters to obtain clean reads [91]. HISAT2 was used for read mapping with default settings [92], and StringTie was used for transcript assembly [93]. Functional annotation was performed using multiple databases, including Nr (NCBI non-redundant protein sequences), Nt (NCBI non-redundant nucleotide sequences), Pfam (Protein family), KOG/COG (Clusters of Orthologous Groups of proteins), Swiss-Prot (manually annotated and reviewed protein sequence database), KO (KEGG Ortholog database), and GO (Gene Ontology). StringTie was also used for quantifying gene expression, using fragments per kilobase of exon per million fragments mapped (FPKM). Differentially expressed genes (DEGs) were identified using DESeq2 (v3.11) with a false discovery rate (FDR) < 0.01 and fold change (FC) ≥ 2 [94]. In addition, chlorophyll fluorescence (Fv/Fm) and relative conductivity were measured. For each biological replicate, three random fields were selected to measure the stomata width-to-length ratio (stomata W/L ratio) and stomata size. Data were analyzed using one-way analysis of variance (ANOVA) in R (v4.3.2) [95].

### 4.9. Weighted Correlation Network Analysis and Gene Network Visualization

Differentially expressed genes (DEGs) with a coefficient of variation (CV) > 0.5 were used to generate co-expression network modules through weighted gene co-expression network analysis (WGCNA) in R [20]. The co-expression modules were identified using the automatic network construction function (blockwiseModules) with default parameters, except for a soft threshold power of 18, TOMtype set to “signed”, mergeCutHeight set to 0.25, and minModuleSize set to 100 [96]. Initial clusters were merged based on eigengenes. The eigengene value for each module was calculated and used to explore associations with the heat stress time, stomatal W/L ratio, stomatal size, Fv/Fm, and relative conductivity. The networks were visualized using Cytoscape (v.3.7.2, USA) [97].

## 5. Conclusions

In summary, we present a high-quality chromosome-level reference genome for the endangered *R. platypodum*, offering new insights into its rocky habitat-related characteristics and the factors contributing to its endangered status. This valuable resource enhances our understanding of rhododendron evolution and supports the conservation of wild species. Additionally, we conducted heat stress experiments on *R. platypodum*, measuring both transcriptomic and physiological responses. Through WGCNA, we identified candidate genes potentially associated with heat stress. These genes hold promise for future applications in the introduction and acclimatization of alpine rhododendrons to lowland environments.

## Figures and Tables

**Table 1:** Comparison of assembly and annotation across 11 different Rhododendron genomes.

	*R. platypodum*	*R. bailiense*	*R. molle*	*R. irroratum*	*R. vialii*	*R. henanense*	*R. ovatum*	*R. griersonianum*	*R. simsii*	*R. delavayi*	*R. williamsianum*
Number of contigs	80	818	34	1549	18	732	2668	67	911	209,969	98,253
N50 of contigs (Mb)	25.64	24.5	44.85	0.69	35.67	2.51	1.24	33.99	2.23	0.06	0.01
Number of scaffolds	46	546	25	1413	13	300	-	48	552	193,091	10,290
N50 of scaffolds (Mb)	49.36	61.88	52.64	51.02	42.05	50.18	41	52.93	36.35	0.64	29.01
Final genome size (Mb)	642.25	923.3	640	701.62	532.73	654.12	549.71	676.81	528.64	695.09	532.29
Complete BUSCOs (%)	98.8	98.3	93.4	96.8	98.5	97	95.3	93.1	93.68	92.8	89
GC content of genome (%)	41	41	40	40	39	41	39	41	39	38	39
LAI	21.13	11.61	-	13.72	-	-	18.32	21.29	17.66	11.17	5.95
Annotation											
Number of predicted genes	36,522	47,567	41,600	49,421	66,464	34,379	43,623	39,510	32,999	32,938	23,559
Average gene length (bp)	6459.24	4146.9	4492.4	4323	4948.8	6393.37	4074	5486.7	5089.22	4434.22	4628
Average CDS length (bp)	1131.89	1372.17	1118.7	1096	1275.7	1208.23	1166	1204.1	1288.73	1153.21	-
Repeat sequences (Mb)	447.19	562.67	349.49	334.77	278.03	417.11	245.48	385.75	250.99	359.87	-
%	69.63	60.96	53.48	47.71	52.19	65.76	44.71	57	47.48	51.77	
LTR-RT length (Mb)	308.19	402.59	277.14	229.97	144.79	321.91	156.03	167.58	89.93	260.53	-
%	47.98	43.61	42.41	32.78	27.18	50.75	28.42	24.76	17.01	37.48	
Source	This manuscript	[22]	[12]	[23]	[24]	[1]	[10]	[4]	[11]	[23]	[25]

## Data Availability

The genome assembly, gene annotations, and raw sequencing data of this study have been deposited in the China National Center for Bioinformation (https://ngdc.cncb.ac.cn) with the BioProject number PRJCA038553.

## Supplementary Materials

The following supporting information can be downloaded at https://www.mdpi.com/article/10.3390/plants14081233/s1, Figure S1: K-mer frequency distribution.; Figure S2: *Rhododendron platypodum* genome-wide all-by-all Hi-C interaction heatmap; Figure S3: Characteristics of the predicted genes in the genomes of *Rhododendron platypodum* compared with *Rhododendron molle*, *Rhododendron simsii*, and *Rhododendron williamsianum*; Figure S4: Bar plot of gene family enrichment analysis for expansion (a) and contraction (b) in *R. platypodum*, classified by biological process; Figure S5: Appearance of *R. platypodum* at different time points after high-temperature stress; Figure S6: Physiological responses of *R. platypodum* after heat stress; Figure S7: PCA of the control group (0d) and experimental groups (1d, 2d, 4d, 6d); Figure S8: GO enrichment analysis of DEGs in 4 treatment groups. (a) 1d vs. 0d, (b) 2d vs. 0d, (c) 4d vs. 0d, (d) 6d vs. 0d; Figure S9: GO enrichment analysis of the blue (a) and turquoise (b) modules; Table S1: Overview of BUSCO assessment; Table S2: Assembly statistics and validation with Hi-C; Table S3: Overview of functional gene annotation statistics; Table S4: Overview of gene annotation; Table S5: Statistics of non-coding RNAs within the *Rhododendron platypodum* genome; Table S6: Comparative genomics of species selection and classification; Table S7: Comparisons of genes and gene families among 16 plant species; Table S8: Expression levels of key genes in two modules based on heat stress duration.
