# Supplementary material for: High-Quality Genome Assembly and Transcriptome of Rhododendron platypodum Provide Insights into Its Evolution and Heat Stress Response
Source: Plants (Basel). 2025 Apr 17;14(8):1233. doi: 10.3390/plants14081233 (PMC12030086; doi:10.3390/plants14081233)
Supplement: Supplementary file 1 [file plants-14-01233-s001.zip › plants-3553801-supplementary.pdf]

Table S1. Overview of BUSCO Assessment

Term	Genes	Percentage (%)
Complete BUSCOs	420	98.8
Complete and single-copy BUSCOs	396	93.2
Complete and duplicated BUSCOs	24	5.6
Fragmented BUSCOs	0	0
Missing BUSCOs	5	1.2
Total BUSCO groups searched	425	100

Table S2. Assembly Statistics and Validation with Hi-c

Assembly Stage	Total (Mb)	Contig Num	Contigs N50 (Mb)	Scaffold Num	Scaffold N50 (Mb)
Initial Assembly	679.98	556	25.64	–	–
Hi-C Assisted Draft Sequence	642.25	80	25.64	–	–
Hi-C Assisted Assembly (Chromosome-anchored)	636.89	47	25.90	13	49.36
Final Assembly	642.25	80	25.64	46	49.36

Table S3. Overview of Functional Gene Annotation Statistics

	Number	Percent (%)
Total	36522	
InterPro	23477	64.28
GO	23075	63.18
KEGG_ALL	30543	83.63
KEGG_KO	10724	29.36
Swissprot	22238	60.89
TrEMBL	31656	86.68
NR	35670	97.67
Annotated	35679	97.69
Unannotated	843	2.31

Table S4. Overview of gene annotation.

Items	Statistics
Gene number	36,522
Average gene length (bp)	6,459.24
Average coding sequence length (bp)	1,131.89
Average exons per gene	4.81
Average exon length (bp)	366.93
Average intron length (bp)	1,193.69

Table S5. Statistics of non-coding RNAs within the *Rhododendron platypodum* genome

Type		Copy	Average length (bp)	Total length (bp)	% of genome
miRNA		87	126.82	11,033	0.00
tRNA		649	75.01	48,681	0.01
rRNA	rRNA	1,964	2,156.10	4,234,584	0.66
	18S	702	1,828.46	1,283,581	0.20
	28S	724	3,990.53	2,889,142	0.45
	5S	538	114.98	61,861	0.01
snRNA	snRNA	480	121.97	58,544	0.01
	CD-box	77	102.34	7,880	0.00
	HACA-box	196	109.95	21,551	0.00
	splicing	207	140.64	29,113	0.00
	scaRNA	0	0.00	0	0.00

Table S6. Comparative Genomics of Species Selection and Classification

	Specific name	Abbreviation name	Order	Family	Genus
TARGET SPECIES	*Rhododendron platypodum*	*R. platypodum*	Ericales	Ericaceae	*Rhododendron*
OTHER SPECIES	*Rhododendron delavayi*	*R. delavayi*	Ericales	Ericaceae	*Rhododendron*
OTHER SPECIES	*Rhododendron henanense*	*R. henanense*	Ericales	Ericaceae	*Rhododendron*
OTHER SPECIES	*Rhododendron griersonianum*	*R. griersonianum*	Ericales	Ericaceae	*Rhododendron*
OTHER SPECIES	*Rhododendron bailiense*	*R. bailiense*	Ericales	Ericaceae	*Rhododendron*
OTHER SPECIES	*Rhododendron irroratum*	*R. irroratum*	Ericales	Ericaceae	*Rhododendron*
OTHER SPECIES	*Rhododendron williamsianum*	*R. williamsianum*	Ericales	Ericaceae	*Rhododendron*
OTHER SPECIES	*Rhododendron molle*	*R. molle*	Ericales	Ericaceae	*Rhododendron*
OTHER SPECIES	*Rhododendron simsii*	*R. simsii*	Ericales	Ericaceae	*Rhododendron*
OTHER SPECIES	*Rhododendron ovatum*	*R. ovatum*	Ericales	Ericaceae	*Rhododendron*
OTHER SPECIES	*Rhododendron vialii*	*R. vialii*	Ericales	Ericaceae	*Rhododendron*
OTHER SPECIES	*Vaccinium duclouxii*	*V. duclouxii*	Ericales	Ericaceae	*Vaccinium*
OTHER SPECIES	*Actinidia eriantha*	*A. eriantha*	Ericales	Actinidiaceae	*Actinidia*
OTHER SPECIES	*Camellia sinensis*	*C. sinensis*	Ericales	Theaceae	*Camellia*
OTHER SPECIES	*Diospyros kaki*	*D. kaki*	Ericales	Ebenaceae	*Diospyros*
OTHER SPECIES	*Camptotheca acuminata*	*C. acuminata*	Cornales	Cornaceae	*Camptotheca*
OTHER SPECIES	*Vitis vinifera*	*V. vinifera*	Vitales	Vitaceae	*Vitis*

Table S7. Comparisons of genes and gene families among 16 plant species

Species	Genes_number	Family_number	Unique_families	Single_copy	Average_genes_per_family
R. platypodum	36,522	19,122	101	344	1.84
V. vinifera	24,978	14,696	294	344	1.65
R. delavayi	32,360	19,140	24	344	1.65
R. henanense	31,098	17,709	67	344	1.72
R. griersonianum	36,540	18,863	124	344	1.89
R. bailiense	47,115	17,745	813	344	2.53
R. irroratum	44,528	19,260	359	344	2.19
R. williamsianum	20,807	14,472	6	344	1.42
R. molle	38,540	19,410	220	344	1.90
R. simsii	32,264	17,072	92	344	1.85
R. ovatum	39,800	17,874	170	344	2.15
R. vialii	29,931	16,557	37	344	1.79
V. duclouxii	41,938	18,166	1,224	344	2.08
A. eriantha	36,215	15,279	310	344	2.30
C. sinensis	31,616	14,892	704	344	1.92
D. lotus	23,873	14,333	203	344	1.63
C. acuminata	27,520	14,708	342	344	1.73

Table S8. Expression levels of key genes in two modules based on heat stress duration

（a）blue module

Gene-ID	Gene-Name	fpkm of 0d	fpkm of 1d	fpkm of 2d	fpkm of 4d	fpkm of 6d
novel.33186	TAR1-A	13.85	8.85	9.52	5.08	36.47
novel.33434	TAR1-A	43.56	29.62	24.75	19.11	77.43
novel.33310	TAR1-A	119.75	80.37	66.21	54.61	216.02
novel.33239	TAR1-A	37.09	29.19	21.93	16.96	61.57
novel.33528	TAR1-A	16.31	14.31	9.35	9.76	38.58
Rpl19100	TAR1-A	21.5	10.61	8.94	9.79	25.85
novel.26413	TAR1-A	14.11	7.33	9.74	4.37	26.18
Rpl31098	TAR1-A	38.39	28.6	23.31	17.56	63.41
novel.33611	TAR1-A	41.51	27.61	23.5	20.49	77.51
novel.17662	TAR1-A	34.2	19.84	17.87	15.05	54.06
Rpl17243	TAR1-A	35.21	24.57	17.2	16.27	54.71
novel.33308	TAR1-A	23.05	21.46	17.15	12.57	67.26
Rpl09654	LRK10	61.91	64.46	27.37	19.34	10.74
Rpl09650	LRK10	43.71	14.3	9.95	6.84	5.56
Rpl09651	LRK10	256.41	171.51	88.73	45.45	37.29
Rpl25584	LRK10	107.58	58.68	40.99	40.53	30.3
Rpl25585	LRK10	63.08	26.12	21.44	20.46	19.81
Rpl35118	LRK10	44.44	20.12	14.2	11.18	11.37
Rpl13213	PHI-1	122.38	38.97	22.39	21.02	42.79
Rpl08113	PHI-1	472.2	161.87	93.3	78.08	97.01
Rpl32918	PHI-1	706.98	324.99	159.1	143.68	299.18
Rpl31755	WRKY24	299.14	151.28	94.81	55.25	57.89
novel.26679	ART2	14.98	22.8	24.58	18.78	79.5
novel.33208	ART2	81.11	57.85	46.2	41.07	139.11
Rpl06882	MIK2	30.68	8.47	8.6	6.85	9.32
Rpl26353	MIK2	143.55	124.21	76.03	74.54	28.87
Rpl06603	VIT_19s0014g04930	24.21	52.19	22.97	9.37	5.36
Rpl34665	2MMP	138.14	50.89	23.47	14.41	13.52

(b) Turquoise module

Gene-ID	Gene-Name	fpkm of 0d	fpkm of 1d	fpkm of 2d	fpkm of 4d	fpkm of 6d
novel.33633	TAR1-A	63.41	41.34	30.09	26.06	112.11
novel.33234	TAR1-A	91.09	67.75	56.67	44.15	173.38
novel.33218	TAR1-A	39.69	29.91	25.98	21.21	74.22
novel.33495	TAR1-A	50.87	35.25	30.5	24.99	93.48
Rpl18761	TAR1-A	13.61	28.58	21.93	8.92	74.76
novel.33412	TAR1-A	84.05	58.52	50.05	40.26	154.76
Rpl36380	TAR1-A	61.45	42.9	33.01	31.49	115.46
Rpl09158	TAR1-A	10.77	9.09	7.31	2.99	24.05
Rpl36054	TAR1-A	38.09	22.35	21.62	17.26	63.09
novel.17578	TAR1-A	8.4	6.13	5.94	4.89	29.43
novel.33303	TAR1-A	48.79	33.4	26.69	22.73	80.13
novel.33619	TAR1-A	39.11	26.14	27.62	21.29	88.51
novel.33568	TAR1-A	79.86	57.44	47.4	37.6	154.25
novel.33095	TAR1-A	71.11	58.8	46.72	35.24	125.61
novel.33224	TAR1-A	8.95	6.54	6.24	4.36	28.63
novel.33354	TAR1-A	26.11	14.65	14.98	8.11	68.56
Rpl23636	TAR1-A	30.39	20.99	19.89	14.19	56.1
novel.33325	TAR1-A	21.63	18.39	19.63	11.49	62.76
novel.33409	TAR1-A	28.96	18.69	18.03	15.73	57.32
Rpl36081	TAR1-A	51.49	36.78	26.91	23.58	92.78
novel.33592	TAR1-A	77.71	57.47	46.67	37.98	149.09
Rpl36191	TAR1-A	28.31	20.47	15.92	15.65	50.7
Rpl18748	TAR1-A	45.17	59.17	45.84	42.88	119.23
novel.33320	TAR1-A	38.79	27.37	22.46	19.25	69.82
Rpl17256	TAR1-A	45.15	34.27	24.29	20.1	86.3
novel.33624	TAR1-A	73.03	50.32	45.49	34.38	133.38
novel.33427	TAR1-A	75.74	53.7	47.03	33.93	146.79
novel.33374	TAR1-A	42.45	30.93	26.65	21.91	72.2
novel.33238	TAR1-A	24.32	14.9	13.83	9.68	46.68
Rpl16767	ABP19A	7.1	1.24	2.33	5.85	41.95
Rpl26057	SAUR32	59.28	32.18	23.67	17.8	18.6
Rpl36069	ART2	25.53	29.5	29.03	16.46	76.62
novel.33431	ART2	49.31	65.67	74.81	49.71	293.22
novel.26626	ART2	1.09	8.53	4.42	6.38	44.26
novel.33477	ART2	69.54	47.5	41.77	31.73	112.28
novel.17667	ART2	41.81	31.75	25.57	17.5	64.52
novel.20085	SDR1	12.31	13.96	37.91	41.2	39.47
novel.20087	SDR1	35.83	7.06	14.35	16.61	21.35
Rpl23803	CRK25	64.39	41.31	27.05	24.06	19.38
Rpl06338	ABCC3	18.42	19.76	7.55	8.03	7.44
Rpl06337	ABCC3	137.3	62.4	33.43	49.93	18.85
Rpl03042	CjBAp12	113.67	433.63	117.44	50.23	45.03

Rpl14832	CjBAp12	9.52	12.05	8.87	1.65	65.36
Rpl34841	At3g26430	1.91	23.97	23.93	20.77	24.33
Rpl34840	At3g26430	1.72	25.83	27.42	27.64	31.3
novel.18270	CYP736A117	33.15	11.88	7.21	9.31	6.88
novel.33504	YLR154W-F	67.47	40.2	36.99	32.67	100.41
novel.26824	YLR154W-F	47.41	30.95	23.5	24.03	71.46
Rpl19250	YLR154W-F	43.57	30.39	29.05	20.17	71.17
novel.33348	YLR154W-F	42.48	28.04	27.7	17.16	68.67
Rpl08764	ATJ11	31.08	254.23	338.18	337.42	409.08
Rpl08112	PHI-1	294.24	115.76	41.67	35.21	47.6
Rpl32917	PHI-1	324.47	150.4	85.16	72.66	109.91
Rpl08114	PHI-1	86.35	35.96	19.78	12.44	31.97

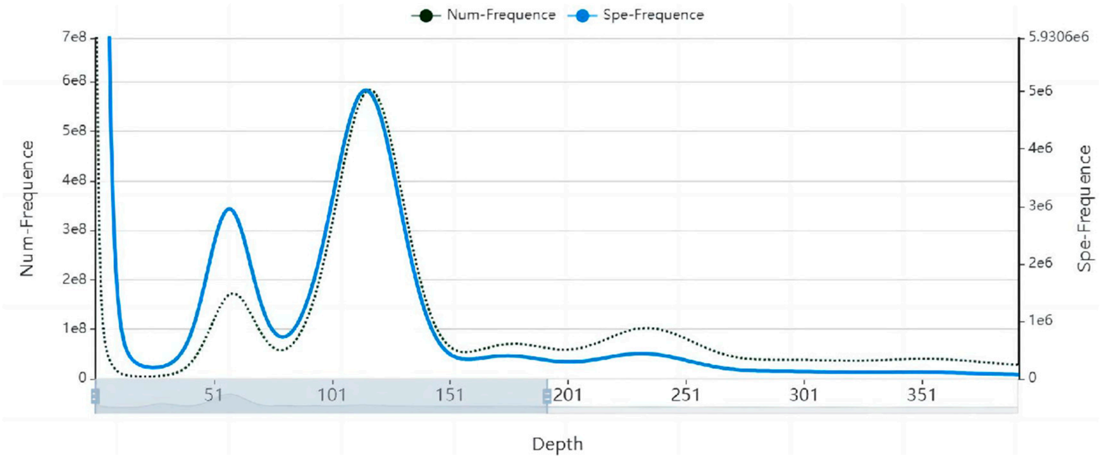

Figure S1. K-mer frequency distribution. According to the result of SURVEY analysis, near depth=114 in the figure is the main peak value, and the genome size calculated by the formula Kmer-number/depth is about 630.62 Mb. The genome heterozygosity is 0.78% and the proportion of repetitive sequences is 58.17%.

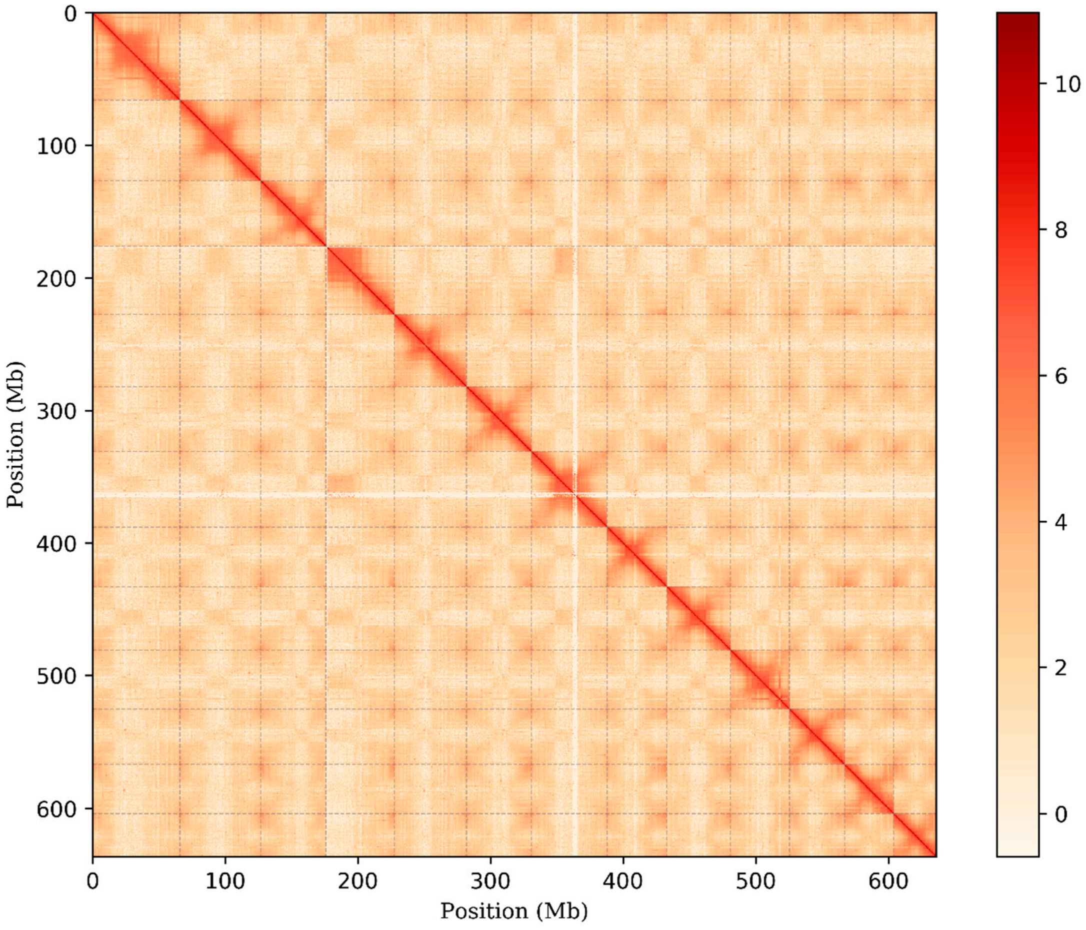

Figure S2. *Rhododendron platypodum* genome-wide all-by-all Hi-C interaction heat map

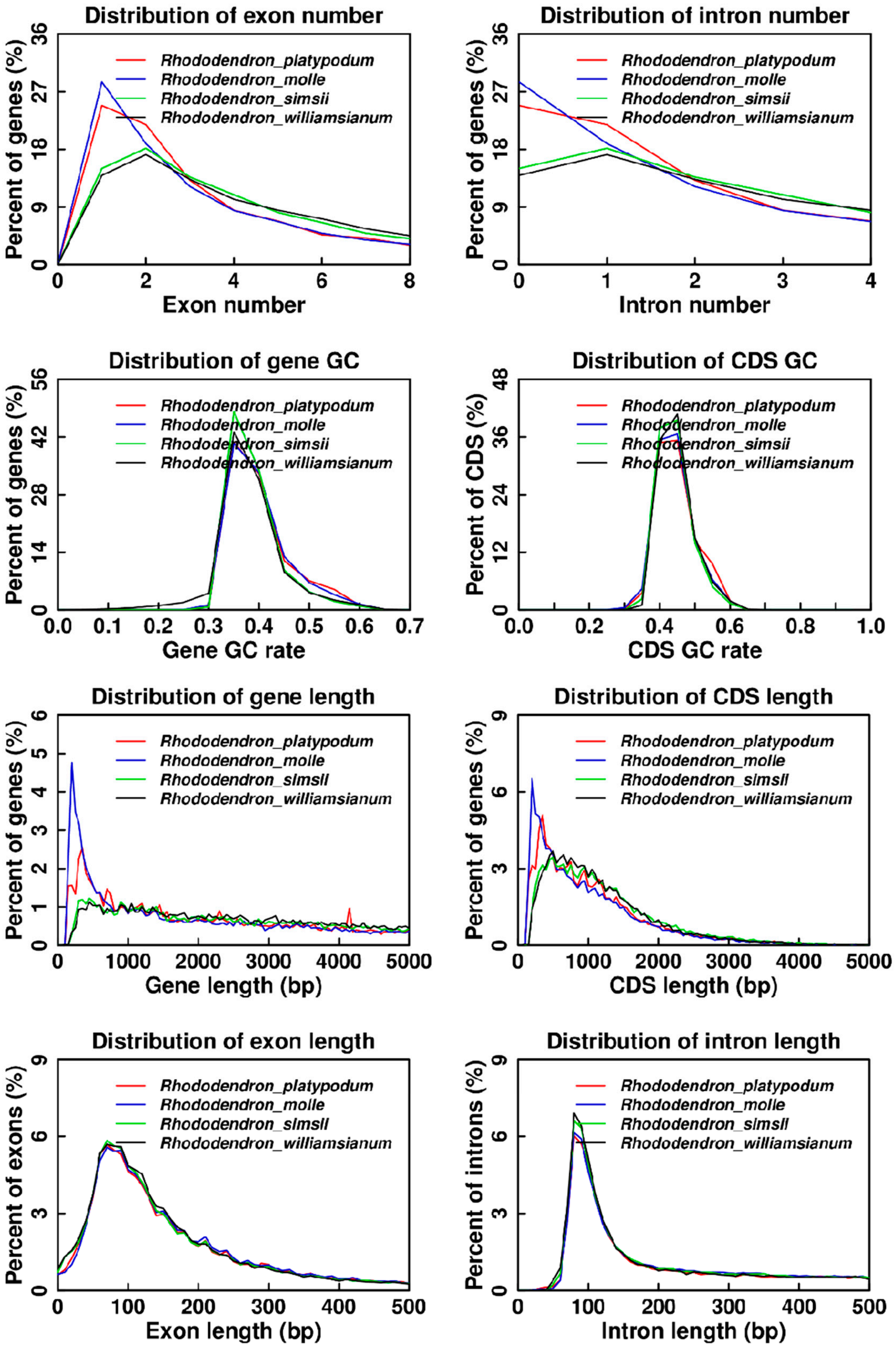

Figure S3. Characteristics of the predicted genes in the genomes of *Rhododendron platypodum* compared with Rhododendron molle, Rhododendron simsii and *Rhododendron williamsianum*.

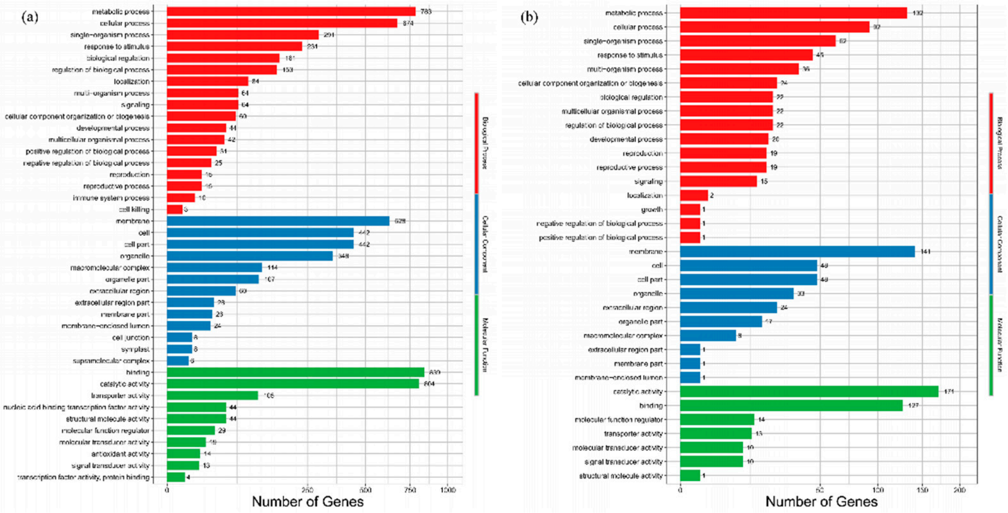

Figure S4. Bar Plot of Gene Family Enrichment Analysis for Expansion (a) and Contraction (b) in *R. platypodum*, Classified by Biological Process

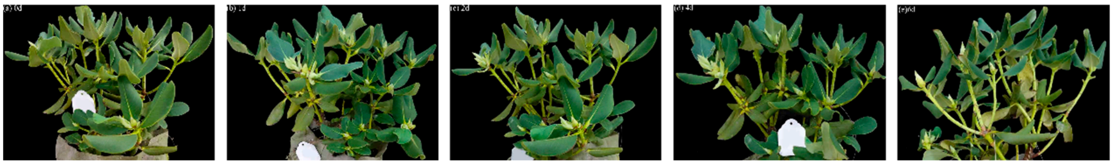

Figure S5. Appearance of *R. platypodum* at Different Time Points after High-Temperature Stress

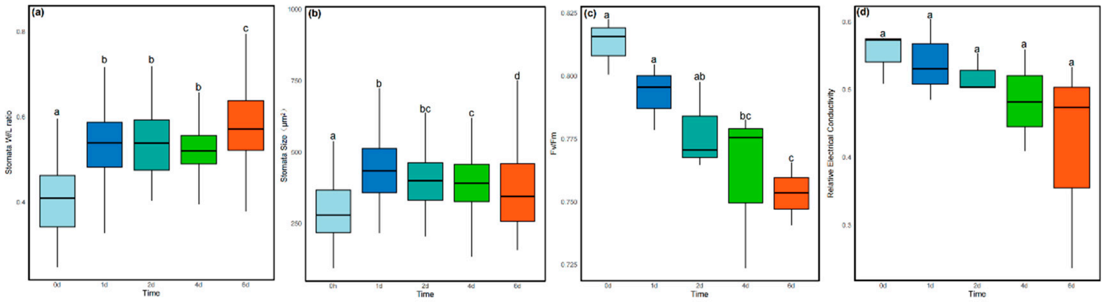

Figure S6. Physiological Responses of *R. platypodum* after Heat Stress

(a) Stomata W/L ratio(b) Stomata size (c) Fv/Fm (d) relatively conductivity

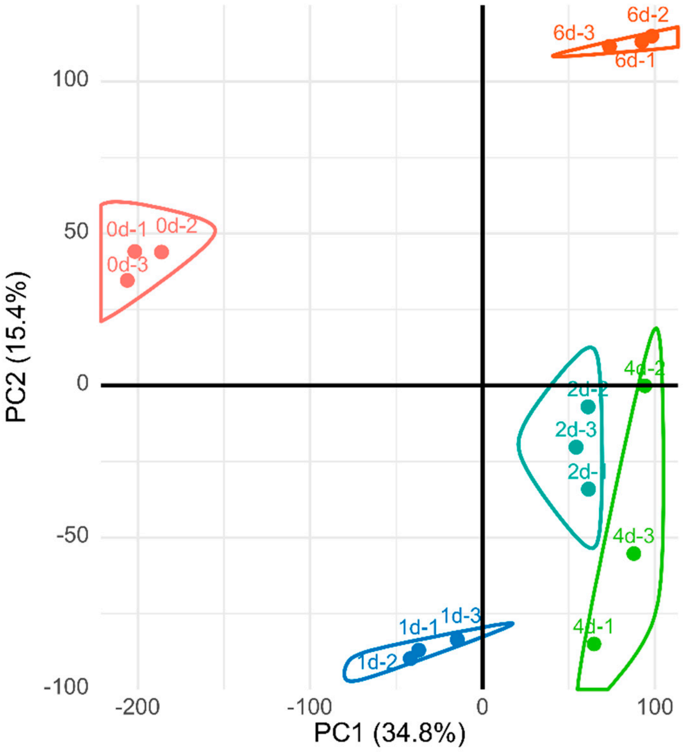

Figure S7. PCA Analysis of the Control Group (0d) and Experimental Groups (1d, 2d, 4d, 6d)

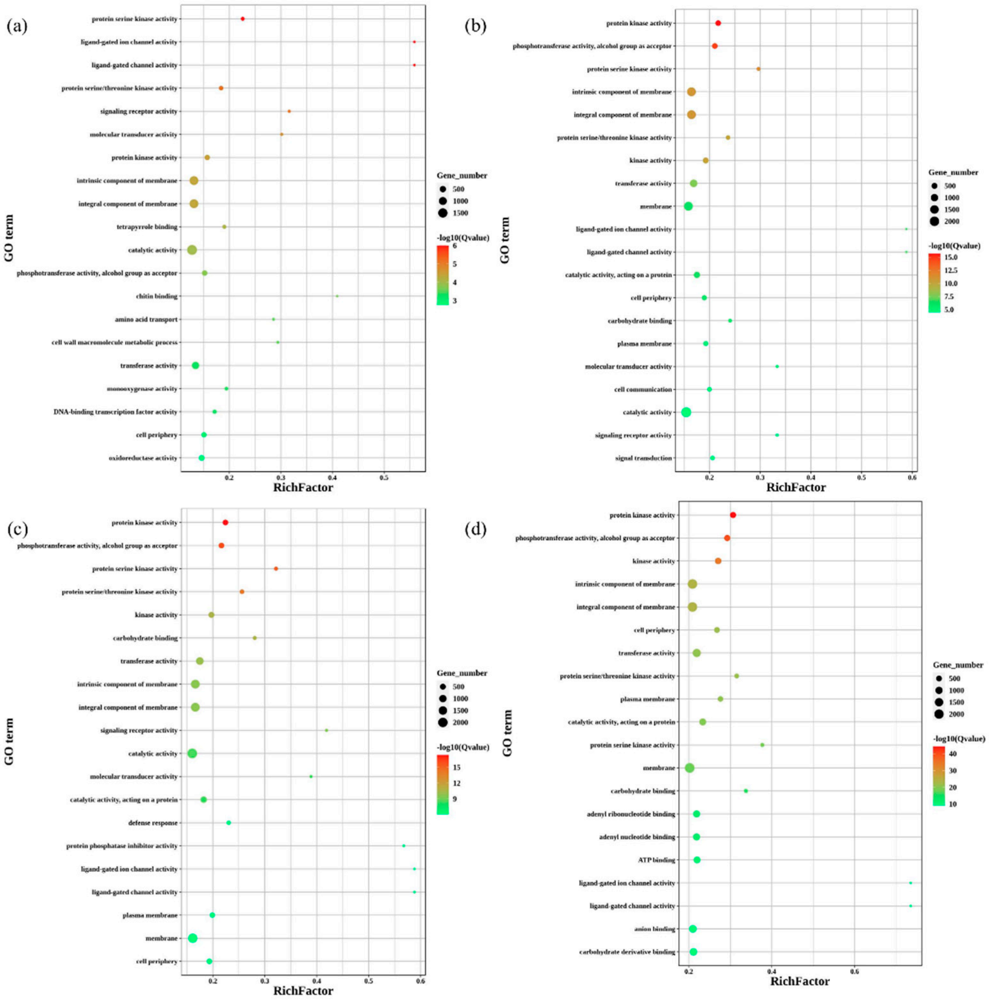

Figure S8. GO Enrichment Analysis of DEGs in 4 Treatment Groups. (a) 1d-vs-0d, (b) 2d-vs-0d, (c) 4d-vs-0d, (d) 6d-vs-0d.

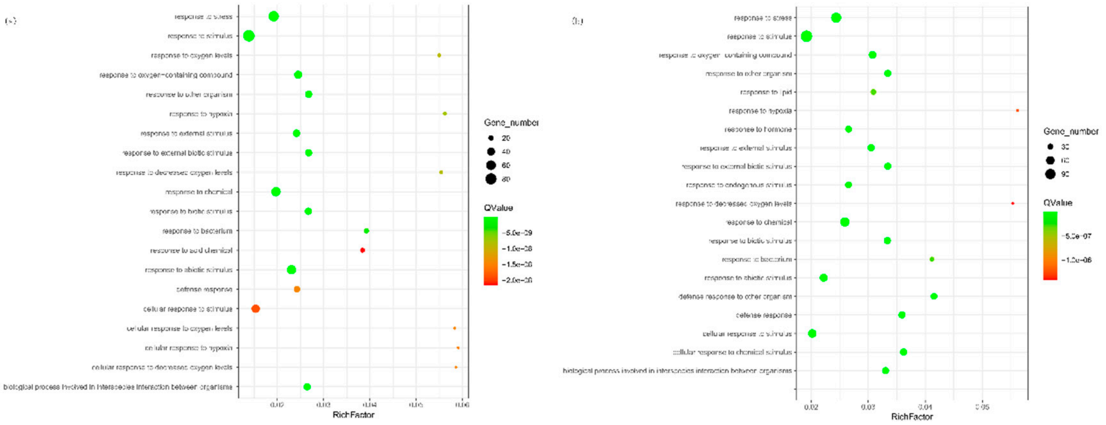

Figure S9. GO Enrichment Analysis of the Blue (a) and Turquoise(b) Modules
